# Continuous variable measurement device independent quantum conferencing with postselection

**DOI:** 10.1038/s41598-022-22251-8

**Published:** 2022-10-15

**Authors:** Alasdair I. Fletcher, Stefano Pirandola

**Affiliations:** Department of Computer Science, University of York, York, YO10 5GH UK

**Keywords:** Other photonics, Quantum information

## Abstract

A continuous variable (CV), measurement device independent (MDI) quantum key distribution (QKD) protocol is analyzed, enabling three parties to connect for quantum conferencing. We utilise a generalised Bell detection at an untrusted relay and a postselection procedure, in which distant parties reconcile on the signs of the displacements of the quadratures of their prepared coherent states. We derive the rate of the protocol under a collective pure-loss attack, demonstrating improved rate-distance performance compared to the equivalent non-post-selected protocol. In the symmetric configuration in which all the parties lie the same distance from the relay, we find a positive key rate over 6 km. Such postselection techniques can be used to improve the rate of multi-party quantum conferencing protocols at longer distances at the cost of reduced performance at shorter distances.

## Introduction

Quantum Key Distribution (QKD) promises provably secure communication^1^ based on fundamental physical principles. Relying on the inability to clone arbitrary quantum states^2^ and by utilising non-orthogonal states or entanglement^3^, two distant parties are able to agree symmetric cryptographic keys, secure against any attack possible within the laws of quantum mechanics. The technology has rapidly matured, advancing from the first proposed protocols based on transmission of discrete single qubit states^4,5^ and proof of principle of experiments to practical deployments over long distances^6–8^ and networks and network protocols enabling multiple users to communicate securely across metropolitan sized areas and beyond^9–11^.

However, whilst QKD offers ultimate security against channel attacks, its practical implementation remains challenging. Many approaches require trusted experimental devices and detectors and therefore suffer from the possibility of so-called *side-channel attacks* against such devices. Fully Device-Independent approaches to QKD are possible, which entirely eliminate such attacks^12–14^ but these are practically limited by low rates and poor distance scaling. Instead Measurement Device Independent (MDI) QKD^15,16^ provides a middle ground, offering higher rates^17^ and various practical implementations^18–20^, by relaxing the assumptions on the protocol to having distant parties send states to a central detector relay which may be controlled by an Eavesdropper (Eve). Malicious behaviour by Eve may be detected by the parties in the reconciliation and parameter estimation stage of the protocol.

Moreover, point-to-point quantum communications are known to be inherently distance limited by the PLOB bound^21^ expressed by the formula $\mathcal {C}=-\log _2(1-\eta )$ with the transmissivity $\eta$ decaying exponentially with distance. Continuous variable (CV) QKD protocols are able to reach rates approaching the PLOB bound, outperforming discrete state protocols; furthermore their experimental implementation is more straightforward^1,22^. Naively, there was thought to be a 3db (corresponding to $\eta =\frac{1}{2}$) loss-limit on CV QKD, however this has since been exceeded with reverse reconciliation, twin-field QKD^23–26^ and postselection techniques. Postselection techniques rely on the fact that even beyond 3db loss there are regions in parameter space in which the rate remains positive^27^. By announcing the absolute values of the quadratures of their prepared coherent states the two end parties are able to select only these regions, reconciling the signs of their quadratures into a key with a positive rate even beyond 3db loss. Such post selection techniques have been implemented experimentally^28^ and have recently been exploited in the MDI setting to extend the maximum distance of two-party CV MDI QKD^29^. Other similar postselection techniques are also possible and have recently been utilised in^30,31^ for discrete modulation CV QKD protocols to improve their distance scaling and tolerance to excess noise.

Whilst such postselection techniques have been shown to improve the distance scaling for typical QKD protocols with two end users; it is also frequently desirable, particularly within a network setting, for multiple users to be able to establish a common secret key. Quantum conferencing^32^ enables the secure distribution of such keys from a single QKD protocol rather than via the composition of multiple bipartite protocols. Such protocols rely on establishing multipartite entangled states between the users such as GHZ states^33^ in the discrete variable case. Quantum conferencing has attracted a great deal of interest and a variety of protocols have been proposed, including MDI protocols with discrete variables^34^, twin field protocols^35–37^, consideration of the effect of finite sized keys^38^ and recently a continuous variable MDI protocol^39^.

In this work, we provide the first demonstration that the same post selection techniques typically applied to two party QKD can also be utilised to increase the effective range at which CV MDI quantum conferencing can occur. We utilise the same generalised Bell detection from the CV MDI quantum conferencing protocol introduced in^39^ to establish multipartite correlations between the user’s variables and a similar postselection procedure to that used in^29^ to extend the effective range of the protocol. Whilst we are restricted by the need to perform numerical integration in large number of dimensions to consider only three parties and pure loss attacks, the protocol presented here is in principle readily extended to *N* users and entangling cloner attacks.

The structure of the paper is as follows: in “Protocol and detector” we introduce the protocol and explain the structure of the detector; “Rate” explains how the rate of the protocol is calculated; “Results” provides results and “Conclusion” is for conclusions.

## Protocol and detector

In this paper, we consider the case of three users undertaking quantum conferencing. The three parties: Alice, Bob and Charlie individually prepare Gaussian modulated coherent states. Each party individually has access to an independent zero-mean Gaussian distribution with standard deviations $\sigma _A,\sigma _B,\sigma _C$ respectively. Each party then draws two independent values from their respective distributions for the value of the *q* and *p* quadratures of their coherent state. They encode the absolute values in the variables $\mathbb {Q}_i$ and $\mathbb {P}_i$ respectively and the signs in $\kappa _i$ and $\kappa _i'$. Thus they prepare coherent states of the form:

1 $$\begin{aligned} \mathinner {|{\alpha _i}\rangle }=\mathinner {|{\frac{1}{2}(\kappa _i\mathbb {Q}_i+\kappa _i'\mathbb {P}_i)}\rangle } \ \ \mathrm {for} \ i=A,B,C \ . \end{aligned}$$

Each state is sent through a lossy channel to the detector which may be attacked by an eavesdropper (Eve). This is modelled as a beamsplitter attack in which Eve inserts a beamsplitter into each channel, storing the outputs in a quantum memory. In a pure loss attack, Eve does not actively inject any state at the beamsplitter and thus each coherent state is instead mixed with the vacuum state $\mathinner {|{0}\rangle }$.

**Figure 1:**
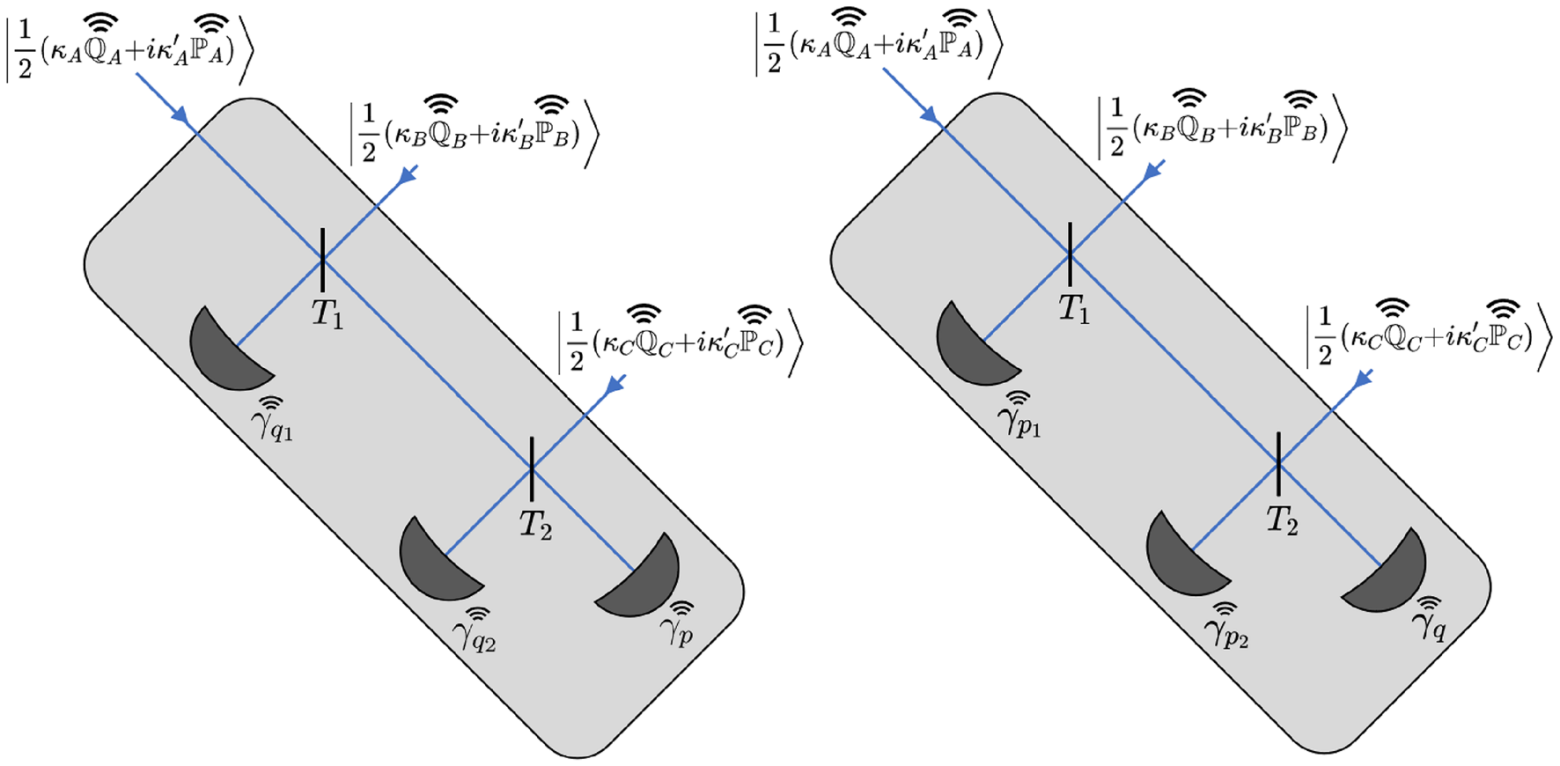
Structure of the detector, demonstrating the two possible orientations. Input modes are mixed by two beamsplitters with transmissivities $T_1=\frac{1}{2}$ and $T_2=\frac{2}{3}$. In the first configuration (pictured left) the states undergo two *q* homodyne detections and one *p* homodyne detection. The parties will attempt reconciliation between $\kappa _A',\kappa _B',\kappa _C'$. In the second orientation (pictured right) the states undergo two *p* homodyne detections and one *q* homodyne detection. In this case the parties attempt reconciliation on $\kappa _A,\kappa _B,\kappa _C$.

**Figure 2:**
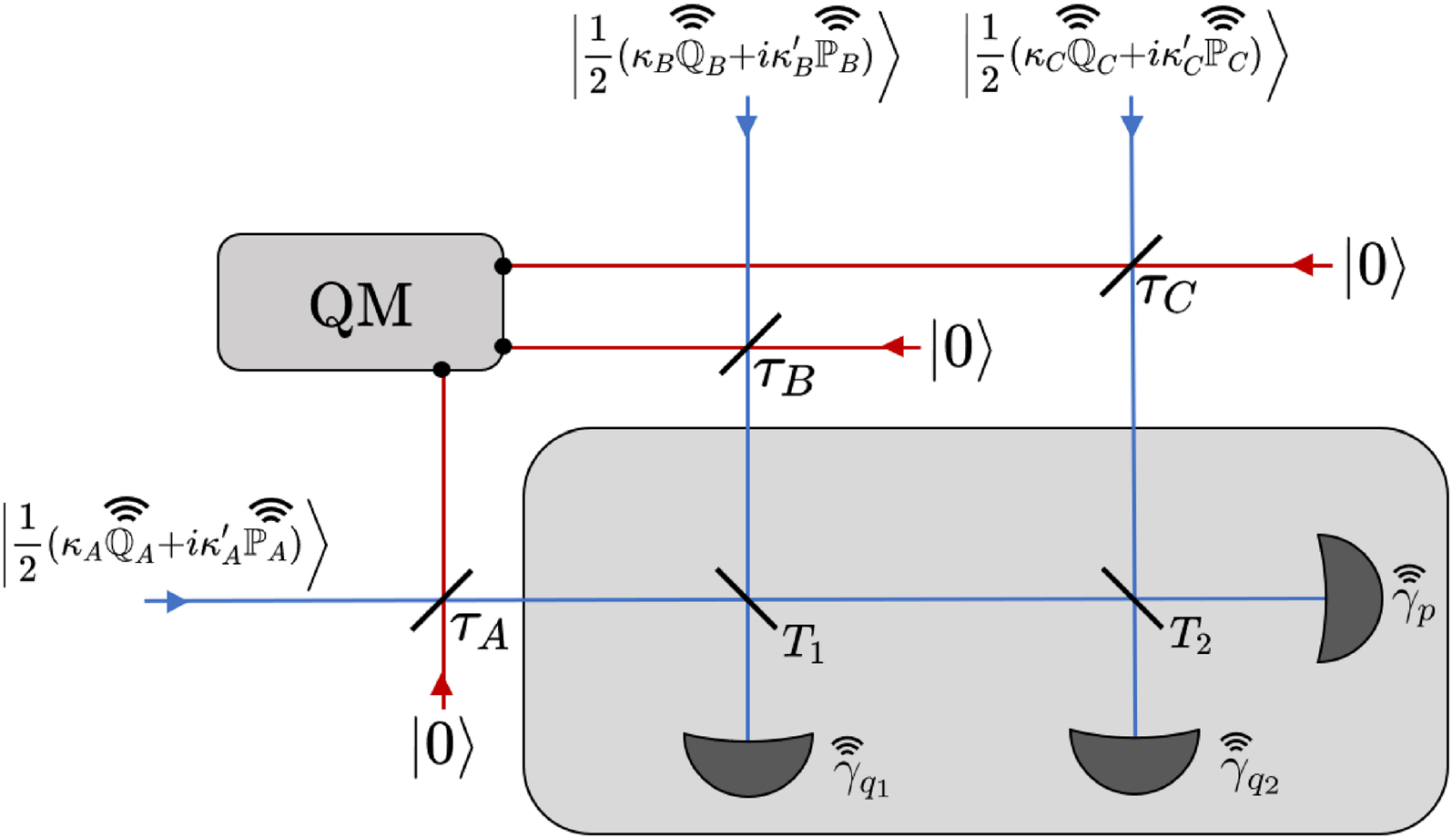
Operation of the detector under a collective pure loss attack. Eve attacks each of the incoming channels by inserting beamsplitters with transmissivities $\tau _A, \tau _B, \tau _C$, which combine the incoming signals with vacuum states $\mathinner {|{0}\rangle }$. Eve stores her output modes in a quantum memory (QM). The remaining modes are mixed in the cascade of beamsplitters and then undergo homodyne detection. The results of the homodyne detections $\gamma _{q_1},\gamma _{q_2},\gamma _{p}$ are publicly announced. Alice, Bob and Charlie also publicly announce the absolute values of the quadratures of their prepared coherent states $\mathbb {Q}_A,\mathbb {Q}_B,\mathbb {Q}_C,\mathbb {P}_A,\mathbb {P}_B,\mathbb {P}_C$. In this configuration the parties attempt to reconcile their values of $\kappa _A',\kappa _B',\kappa _C'$.

The structure of the detector is illustrated in Fig. 1 and was devised in^39^ to perform a generalised Bell detection on the incoming coherent states. It is comprised of a cascade of beamsplitters, each having transmissivity $T_i=\frac{i}{i+1}$. In the case of three parties, which we consider, this corresponds to $T_1=1/2$ and $T_2=2/3$. The beamsplitters are followed by two *q*  (*p*) homodyne detections and a final homodyne detection in the *p*  (*q*) quadrature and the results of all the measurements are publicly broadcast. Operated correctly, in the entanglement based representation^1^ the detector has the effect of projecting the Alice-Bob-Charlie state into a symmetric state with GHZ-like correlations between each parties state^39^. The two possible configurations are switched between randomly and are announced during the basis reconciliation stage of the protocol. If the first configuration (two *q* and one *p* detection) was selected, the parties will attempt to reconcile their values of $\kappa _A',\kappa _B',\kappa _C'$ into a secure key. Conversely, if the second configuration is utilised, the parties will attempt to reconcile their values of $\kappa _A,\kappa _B,\kappa _C$. At this point each party reveals their values of $\mathbb {Q}_i$ and $\mathbb {P}_i$, and publicly broadcasts them to every other user. Using their knowledge of $\mathbb {Q}_i$ and $\mathbb {P}_i$, the parties can perform postselection, only retaining instances of the protocol where their mutual information exceeds Eve’s Holevo information. Figure 2 depicts the protocol being performed under the pure loss attack assumed throughout this paper.

## Rate

We first sketch the method used to determine the rate. At the end of the protocol the parties perform pairwise reconciliation between $\kappa _A,\kappa _B,\kappa _C$ or $\kappa _A',\kappa _B',\kappa _C'$ depending on the orientation of the detector. In the asymptotic limit of a large number of uses the rate of the protocol is given by:

2 $$\begin{aligned} R_{ij}=I_{ij}-\chi \end{aligned}$$

where $I_{ij}$ is the binary mutual information between the sign variables $\kappa _i$ and $\kappa _j$ or $\kappa '_i$ and $\kappa _j'$. $\chi$ is the Holevo information. The mutual information can be found by utilising Bayes’ Theorem and the distribution of measurement outcomes as detailed in “Mutual information”. The Holevo information is calculated by carefully considering Eve’s state at the end of the protocol as explained in “Holevo bound”. Additionally, since we ultimately wish to perform postselection to increase the performance of the protocol we work with *single-point* versions of the above quantities $\tilde{I}_{ij}$ and $\tilde{\chi }$ which are the values conditioned upon the quadratures and measurement outcome. To this end we start by considering the initial covariance matrix of the Alice–Bob–Charlie–Eve system, which is given by:

3 $$\begin{aligned} \mathbf {V}_{ABCE}=\mathbf {I}_A\oplus \mathbf {I}_B\oplus \mathbf {I}_C\oplus \mathbf {V}_{E} \end{aligned}$$

where $\mathbf {I}$ is the two-by-two identity matrix and for a pure loss attack $\mathbf {V}_{E}=\mathbf {I}\oplus \mathbf {I}\oplus \mathbf {I}$. The mean value of the Alice–Bob–Charlie system is:

4 $$\begin{aligned} {\bar{\mathbf {x}}}_{ABC}=(\kappa _A\mathbb {Q}_A,\kappa _A'\mathbb {P}_A,\kappa _B\mathbb {Q}_B,\kappa _B'\mathbb {P}_B,\kappa _C\mathbb {Q}_C,\kappa _C'\mathbb {P}_C)^T \ . \end{aligned}$$

The mean value of Eve’s system is zero. After propagation through the detector’s array of beamsplitters and the homodyne detections, the distribution of measurement outcomes is given by:

5 $$\begin{aligned} p(\gamma _p|\kappa _A',\kappa _B',\kappa _C',\mathbb {P}_A,\mathbb {P}_B,\mathbb {P}_C)=\frac{1}{\sqrt{2\pi v_p}}\mathrm {exp}\bigg (\frac{-(\gamma _p-\bar{p})^2}{2} \bigg ) \end{aligned}$$

where

6 $$\begin{aligned} \bar{p}=\sqrt{T_1T_2\tau _A}\ \kappa _A' \mathbb {P}_A+\sqrt{(1-T_1)T_2\tau _B} \ \kappa _B' \mathbb {P}_B&+ \sqrt{(1-T_2)\tau _C} \ \kappa _C'\mathbb {P}_C \ . \end{aligned}$$

We have implicitly removed the conditioning on the modulus and absolute value of the *q* quadratures from the notation as there is no dependence upon them. Similarly for the opposite detector configuration:

7 $$\begin{aligned} p(\gamma _q|\kappa _A,\kappa _B,\kappa _C,\mathbb {Q}_A,\mathbb {Q}_B,\mathbb {Q}_C)=\frac{1}{\sqrt{2\pi v_q}}\mathrm {exp}\bigg ( \frac{-(\gamma _{q}-\bar{q})^2}{2}\bigg ) \end{aligned}$$

where

8 $$\begin{aligned} \bar{q}=\sqrt{T_1T_2\tau _A}\ \kappa _A \mathbb {Q}_A+\sqrt{(1-T_1)T_2\tau _B} \ \kappa _B \mathbb {Q}_B + \sqrt{(1-T_2)\tau _C} \ \kappa _C\mathbb {Q}_C \ . \end{aligned}$$

Finally, we have implicitly assumed throughout that the homodyne detectors have perfect efficiency.

### Mutual information

We first introduce the following compact notation $\varvec{\kappa '}=(\kappa _A',\kappa _B',\kappa _C')$; $\pmb {\mathbb {P}}=(\mathbb {P}_A,\mathbb {P}_B,\mathbb {P}_C)$, $\varvec{\kappa }^{\prime }_{\backslash \, A}=(\kappa _B',\kappa _C')$ which simplifies the following expressions. Let us recall the definition of the single point mutual information between the two binary variables $\kappa _i'$ and $\kappa _j'$. This is clearly just the mutual information conditioned on the announced variables $\gamma _p$ and $\pmb {\mathbb {P}}$:

9 $$\begin{aligned} {\tilde{I}}_{ij}=H_{\kappa _{i}'|\pmb {\mathbb {P}},\gamma _p}-\sum _{\kappa _{j}'}p(\kappa _{j}'|~\pmb {\mathbb {P}},\gamma _p)~H_{\kappa _{i}'|\kappa _{j}'\pmb {\mathbb {P}},\gamma _{p}} \end{aligned}$$

where *H* is the binary entropy so that:

10 $$\begin{aligned} H_{\kappa _{i}'|\pmb {\mathbb {P}},\gamma _p}=-p(\kappa _{i}'|\pmb {\mathbb {P}},\gamma _p)\log _2\big (p(\kappa _{i}'|\pmb {\mathbb {P}},\gamma _p)\big )-\big (1-p(\kappa _{i}'|\pmb {\mathbb {P}},\gamma _p)\big )\log _2\big (1-p(\kappa _{i}'|\pmb {\mathbb {P}},\gamma _p)\big )\, \end{aligned}$$

and

11 $$\begin{aligned} H_{\kappa _{i}'|\kappa _{j}',\pmb {\mathbb {P}},\gamma _p}=-p(\kappa _{i}'|\kappa _{j}',\pmb {\mathbb {P}},\gamma _p)\log _2(p(\kappa _{i}'|\kappa _{j}',\pmb {\mathbb {P}},\gamma _p))-\Big (1-p(\kappa _{i}'|\kappa _j',\pmb {\mathbb {P}},\gamma _p)\Big )\log _2\Big (1-p(\kappa _{i}'|\kappa _j',\pmb {\mathbb {P}},\gamma _p)\Big ) \ . \end{aligned}$$

From the symmetry of the detector we have $I_{AB}=I_{AC}=I_{BC}$ and for simplicity we consider only $I_{AB}$ from this point onwards. Using Eq. (5) and Bayes’ theorem we first calculate the probability of positive and negative values for $\kappa _A'$ conditioned on $\kappa _B'$,$\kappa _C'$, the magnitudes of the *p* quadratures $\pmb {\mathbb {P}}$ and the measurement outcome $\gamma _{p}$:

12 $$\begin{aligned} p(\kappa _A'|\varvec{\kappa }^{\prime }_{\backslash \, A} ,\pmb {\mathbb {P}},\gamma _p)=\frac{p(\gamma _p|\varvec{\kappa }',\pmb {\mathbb {P}})~p(\kappa '_A|\varvec{\kappa }^{\prime }_{\backslash \, A},\pmb {\mathbb {P}})}{p(\gamma _p|\varvec{\kappa }^{\prime }_{\backslash \, A},\pmb {\mathbb {P}})} \end{aligned}$$

Noting that,

13 $$\begin{aligned} p(\gamma _p|\varvec{\kappa }^{\prime }_{\backslash \, A},\pmb {\mathbb {P}})=\sum _{\kappa '_A}p(\gamma _p|\varvec{\kappa }',\pmb {\mathbb {P}})~p(\kappa '_A|\varvec{\kappa }^{\prime }_{\backslash \, A},\pmb {\mathbb {P}}) \end{aligned}$$

and $p(\kappa '_A|\varvec{\kappa }^{\prime }_{\backslash \, A},\pmb {\mathbb {P}})=1/2$ we reach:

14 $$\begin{aligned} p(\kappa _A'|\varvec{\kappa }^{\prime }_{\backslash \, A},\pmb {\mathbb {P}},\gamma _p)=\frac{p(\gamma _p|\varvec{\kappa }',\pmb {\mathbb {P}})}{\sum _{\kappa _A'}p(\gamma _p|\varvec{\kappa }',\pmb {\mathbb {P}})} . \end{aligned}$$

We may then remove the conditioning on $\kappa '_C$ to find $p(\kappa _A'|\kappa _B', \pmb {\mathbb {P}},\gamma _p)$ for the second term in the single point mutual information.

15 $$\begin{aligned} p(\kappa _A'|\kappa _B' , \pmb {\mathbb {P}},\gamma _p)=\sum _{\kappa _C'}p(\kappa _A'|\varvec{\kappa }^{\prime }_{\backslash \, A},\pmb {\mathbb {P}}\gamma _p)~p(\varvec{\kappa }^{\prime }_{\backslash \, b}|\kappa _B',\pmb {\mathbb {P}}), \end{aligned}$$

so that we may write

16 $$\begin{aligned} p(\kappa _A'|\kappa _B' , \pmb {\mathbb {P}})=\frac{\sum _{\kappa _C'}p(\gamma _p|\varvec{\kappa }',\pmb {\mathbb {P}})}{\sum _{\kappa _A'\kappa _C'}p(\gamma _p|\varvec{\kappa },'\pmb {\mathbb {P}})}. \end{aligned}$$

Similarly to further remove the dependence from $\kappa '_B$:

17 $$\begin{aligned} p(\kappa '_A|\pmb {\mathbb {P}},\gamma _p)=\frac{\sum _{\kappa '_B\kappa _C'}p(\gamma _p|\varvec{\kappa }',\pmb {\mathbb {P}})}{\sum _{\kappa '_A\kappa '_B\kappa _C'}p(\gamma _p|\varvec{\kappa }',\pmb {\mathbb {P}})}. \end{aligned}$$

By the same approach we can also find $p(\kappa _B'|\pmb {\mathbb {P}},\gamma _p)$, enabling the sum in Eq. (9) to be taken. Finally in order to take the integral over the single point mutual information we require the probability of all the variables

18 $$\begin{aligned} p(\gamma _p,\pmb {\mathbb {P}})=\sum _{\varvec{\kappa '}} p(\gamma _p|\varvec{\kappa '}\pmb {\mathbb {P}})~p(\kappa _A' \mathbb {P}_A)~p(\kappa _B' \mathbb {P}_B)~p(\kappa _C' \mathbb {P}_C). \end{aligned}$$

### Holevo bound

At the end of the protocol Eve is left with the state $\hat{\rho }_{\mathfrak {E}|\pmb {\mathbb {P}},\gamma _p}$ which is her total state conditioned on the announced absolute values of the *p* quadratures $\pmb {\mathbb {P}}$ and the measurement outcome $\gamma _p$. This state is a convex combination of pure Gaussian states corresponding to given values of $\kappa _A',\kappa _B',\kappa _C'$ and hence Eve’s total state may be written:

19 $$\begin{aligned} \hat{\rho }_{\mathfrak {E}|\pmb {\mathbb {P}},\gamma _p}=\sum _{\varvec{\kappa '}}p(\varvec{\kappa '}|\pmb {\mathbb {P}},\gamma _p)~\hat{\rho }_{\mathfrak {E}|\varvec{\kappa '},\pmb {\mathbb {P}},\gamma _p}. \end{aligned}$$

It is important to note that whilst the conditional states, $\hat{\rho }_{\mathfrak {E}|\varvec{\kappa '},\pmb {\mathbb {P}},\gamma _p}$, are pure and Gaussian the total state, $\hat{\rho }_{\mathfrak {E}|\pmb {\mathbb {P}},\gamma _p}$ is not, which complicates our analysis. Nonetheless, assuming that Eve performs a collective attack on the protocol the relevant quantity to calculate is the Holevo information $\chi$. We can again write this as a single point quantity in the following way.

20 $$\begin{aligned} \tilde{\chi }(\mathfrak {E}:\kappa _{i}'|~\pmb {\mathbb {P}},\gamma _p)=S(\hat{\rho }_{\mathfrak {E}|\pmb {\mathbb {P}},\gamma _p})-S(\hat{\rho }_{\mathfrak {E}|\kappa _{i}',\pmb {\mathbb {P}},\gamma _p}) \end{aligned}$$

where $\tilde{\chi }(\mathfrak {E}:\kappa _{i}'|~\pmb {\mathbb {P}},\gamma _p)$ is the single point Holevo information and *S* is the von Neumann entropy which we recall is calculated from the eigenvalues $\{\lambda _i\}$ of a density matrix $\hat{\rho }$ by:

21 $$\begin{aligned} S(\hat{\rho })=-\sum _i \lambda _i\log _2(\lambda _i). \end{aligned}$$

First let us write the conditional states $\hat{\rho }_{\mathfrak {E}|\varvec{\kappa '}\pmb {\mathbb {P}}\gamma _p}$ as:

22 $$\begin{aligned} \hat{\rho }_{\mathfrak {E}|\varvec{\kappa '}\pmb {\mathbb {P}}\gamma _p}=\mathinner {|{\mathfrak {E}^{\pmb {\mathbb {P}},\gamma _p}_{\kappa _{A}'\kappa _{B}'\kappa _{C}'}}\rangle }\mathinner {\langle {\mathfrak {E}^{\pmb {\mathbb {P}},\gamma _p}_{\kappa _{A}'\kappa _{B}'\kappa _{C}'}}|} \end{aligned}$$

We consider the matrix of overlaps *O* of this state for all the combinations of $\kappa _A',\kappa _B',\kappa _C'$.

23 $$\begin{aligned} O=&\begin{pmatrix} 1 &{} C &{} B &{} BC &{} A &{} AC &{} AB &{} ABC \\ C &{} 1 &{} BC &{} B &{} AC &{} A &{} ABC &{} AB \\ B &{} BC &{} 1 &{} C &{} AB &{} ABC &{} A &{} AC \\ BC &{} B &{} C &{} 1 &{} ABC &{} AB &{} AC &{} A \\ A &{} AC &{} AB &{} ABC &{} 1 &{} C &{} B &{} BC \\ AC &{} A &{} ABC &{} AB &{} C &{} 1 &{} BC &{} B \\ AB &{} ABC &{} A &{} AC &{} B &{} BC &{} 1 &{} C \\ ABC &{} AB &{} AC &{} A &{} BC &{} B &{} C &{} 1 \end{pmatrix} \begin{matrix} (-1 &{} -1 &{}-1) \\ (-1 &{}-1 &{} 1) \\ (-1&{}1&{}-1) \\ (-1&{}1&{}1) \\ (1&{}-1&{}-1) \\ (1&{}-1&{}1) \\ (1&{}1-&{}1) \\ (1&{}1&{}1) \end{matrix} \end{aligned}$$

The values in the far column denote the row values of $\kappa _A',\kappa _B',\kappa _C'$. The columns may be similarly labelled. *O* is clearly separable as:

24 $$\begin{aligned} O= \begin{pmatrix} 1 &{} A \\ A &{} 1 \end{pmatrix} \otimes \begin{pmatrix} 1 &{} B \\ B &{} 1 \end{pmatrix} \otimes \begin{pmatrix} 1 &{} C \\ C &{} 1 \end{pmatrix} \end{aligned}$$

which implies:

25 $$\begin{aligned} \mathinner {|{\mathfrak {E}^{\pmb {\mathbb {P}},\gamma _p}_{\kappa _{A}'\kappa _{B}'\kappa _{C}'}}\rangle }=\mathinner {|{\mathfrak {E'}_{\kappa _{A}'}^{\pmb {\mathbb {P}},\gamma _p}}\rangle } \otimes \mathinner {|{\mathfrak {E}^{\pmb {\mathbb {P}},\gamma _p}_{\kappa _{B}'}}\rangle }\otimes \mathinner {|{\mathfrak {E'}_{\kappa _{C}'}^{\pmb {\mathbb {P}},\gamma _p}}\rangle }. \end{aligned}$$

Each of these states lies in a two-dimensional Hilbert space. Using *x* to index the parties *A*, *B*, *C* we may expand the states as:

26 $$\begin{aligned} \mathinner {|{\mathfrak {E}_{\kappa _{i}=-1}^{\pmb {\mathbb {P}},\gamma _p}}\rangle }=c_{0}\mathinner {|{\Phi ^{({x})}_{0}}\rangle }+c_{1}\mathinner {|{\Phi ^{(x)}_{1}}\rangle } \end{aligned}$$

27 $$\begin{aligned} \mathinner {|{\mathfrak {E}_{\kappa _{i}=1}^{\pmb {\mathbb {P}},\gamma _p}}\rangle }=c_{0}\mathinner {|{\Phi _{0}^{(x)}}\rangle }-c_{1}\mathinner {|{\Phi _{1}^{(x)}}\rangle } \end{aligned}$$

and find the following relation for the coefficients:

28 $$\begin{aligned} |c^{(x)}_{0}|^2=\frac{1}{2}(1+X) \end{aligned}$$

29 $$\begin{aligned} |c^{(x)}_{1}|^2=\frac{1}{2}(1-X) \end{aligned}$$

where *X* labels the corresponding values *A*, *B*, *C* from Eq. (23). For two Gaussian states with the same covariance matrix $\mathbf {V}$ and mean values $\bar{\mathbf {x}}_{1}$ and $\bar{\mathbf {x}}_{2}$ the following relation holds^40^:

30 $$\begin{aligned} \mathrm {Tr}(\hat{\rho }_{1}\hat{\rho }_{2})={\mathrm {exp}}\bigg (-\frac{1}{4}({\bar {\mathbf{x}}}_1-{\bar {\mathbf{x}}}_2)\mathbf {V}^{-1}({\bar {\mathbf{x}}}_1-{\bar {\mathbf{x}}}_2)\bigg ) \end{aligned}$$

which we use to calculate

31 $$\begin{aligned} A=\langle \mathfrak {E}_{\kappa _{A}=-1}^{\pmb {\mathbb {P}},\gamma _p}|\mathfrak {E}_{\kappa _{A}=1}^{\pmb {\mathbb {P}},\gamma _p}\rangle , \end{aligned}$$

32 $$\begin{aligned} B=\langle \mathfrak {E}_{\kappa _{B}=-1}^{\pmb {\mathbb {P}},\gamma _p}|\mathfrak {E}_{\kappa _{B}=1}^{\pmb {\mathbb {P}},\gamma _p}\rangle , \end{aligned}$$

33 $$\begin{aligned} C=\langle \mathfrak {E}_{\kappa _{C}=-1}^{\pmb {\mathbb {P}},\gamma _p}|\mathfrak {E}_{\kappa _{C}=1}^{\pmb {\mathbb {P}},\gamma _{p}}\rangle . \end{aligned}$$

We are now able to give $\hat{\rho }_{\mathfrak {E}|\pmb {\mathbb {P}},\gamma _p}$ in the $\{\mathinner {|{\Phi _{0}^{(A)}}\rangle },\mathinner {|{\Phi _{1}^{(A)}}\rangle }\}\otimes \{\mathinner {|{\Phi _{0}^{(B)}}\rangle },\mathinner {|{\Phi _{1}^{(B)}}\rangle }\}\otimes \{\mathinner {|{\Phi _{0}^{(C)}}\rangle },\mathinner {|{\Phi _{1}^{(C)}}\rangle }\}$ basis. Describing the row position with the binary string (*i*, *j*, *k*) and similarly the column position with $(i',j',k')$ each component of the density matrix can be calculated by:

34 $$\begin{aligned} ({\hat{\rho }}_{\mathfrak {E}|\pmb {\mathbb {P}}\gamma _p})_{(ijk)(i'j'k')}=\sum _{\varvec{\kappa '}}p(\varvec{\kappa '}|\pmb {\mathbb {P}},\gamma _p)\langle \Phi _{i}^{(A)}|\mathfrak {E}^{\pmb {\mathbb {P}},\gamma _p}_{\kappa _A}\rangle \langle \mathfrak {E}^{\pmb {\mathbb {P}},\gamma _p}_{\kappa _A}|\Phi _{i'}^{(A)}\rangle \langle \Phi _{j}^{(B)}|\mathfrak {E}^{\pmb {\mathbb {P}},\gamma _p}_{\kappa _B}\rangle \langle \mathfrak {E}^{\pmb {\mathbb {P}},\gamma _p}_{\kappa _B}|\Phi _{j'}^{(2)}\rangle \langle \Phi _{k}^{(C)}|\mathfrak {E}^{\pmb {\mathbb {P}},\gamma _p}_{\kappa _C'}\rangle \langle \mathfrak {E}^{\pmb {\mathbb {P}},\gamma _p}_{\kappa _C'}|\Phi _{k'}^{(C)}\rangle . \end{aligned}$$

By calculating the following inner products:

35 $$\begin{aligned} \langle \Phi ^{(x)}_0|\mathfrak {E}^{\pmb {\mathbb {P}},\gamma _p}_{\kappa _x=-1}\rangle =c^{(x)}_0 \end{aligned}$$

36 $$\begin{aligned} \langle \Phi ^{(i)}_0|\mathfrak {E}^{\pmb {\mathbb {P}},\gamma _p}_{\kappa _x=1}\rangle =c^{(x)}_0 \end{aligned}$$

37 $$\begin{aligned} \langle \Phi ^{(i)}_1|\mathfrak {E}^{\pmb {\mathbb {P}},\gamma _p}_{\kappa _x=-1}\rangle =c^{(x)}_1 \end{aligned}$$

38 $$\begin{aligned} \langle \Phi ^{(i)}_1|\mathfrak {E}^{\pmb {\mathbb {P}},\gamma _p}_{\kappa _x=1}\rangle =-c^{(x)}_1 \end{aligned}$$

we can therefore immediately find the diagonal components of the density matrix:

39 $$\begin{aligned} (\hat{\rho }_{\mathfrak {E}|\pmb {\mathbb {P}},\gamma _p})_{(ijk)(ijk)}=|c_{i}^{(A)}|^2 \ |c_{j}^{(B)}|^2 \ |c_{k}^{(C)}|^2. \end{aligned}$$

The off diagonal terms are given by:

40 $$\begin{aligned} (\hat{\rho }_{\mathfrak {E}|\pmb {\mathbb {P}},\gamma _p})_{(ijk)(i'j'k')}=c_{i}^{(A)}\big (c_{i'}^{(A)}\big )^*c_{j}^{(B)}\big (c_{j'}^{(B)}\big )^*c_{k}^{(C)}\big (c_{k'}^{(C)}\big )^*\Lambda ({i,j,k,i',j',k'}) \end{aligned}$$

where $\Lambda (i,j,k,i',j',k')$ is given by

41 $$\begin{aligned} \Lambda (i,j,k,i',j',k')=\sum _{\varvec{\kappa '}}(-1)^{f(\kappa _A')|i-i'|+f(\kappa _B')|j-j'|+f(\kappa _C')|k-k'|}~p(\varvec{\kappa '}|\pmb {\mathbb {P}},\gamma _p) \end{aligned}$$

where *f* is a function such that $f(\kappa _i=-1)=0$ and $f(\kappa _i=1)=1$. We therefore have all the components of $\hat{\rho }_{\mathfrak {E}|\pmb {\mathbb {P}},\gamma _p}$ from which we may numerically find the eigenvalues and compute the first term in the Holevo bound (Eve’s conditional output state following the protocol $\hat{\rho }_{\mathfrak {E}|\pmb {\mathbb {P}},\gamma _p}$ has dimension $2^N$. Therefore for $N\ge 3$, including the tri-partite case considered in this paper, the eigenvalues of this state cannot be given in closed form. Therefore the entropy of the state and consequently the single point Holevo information $\tilde{\chi }$ can only be evaluated numerically for given values of the protocol’s parameters. This greatly complicates numerical integration in Eq. (47) as no explicit expression for the single point rate can be given. It is for this reason that our analysis is limited to pure loss attacks and three users, even though the analysis is readily extended to an arbitrary number of users and entangling cloner attacks.). For the second term in the Holevo bound we need Eve’s state conditioned on $\kappa _{A}$. If $\kappa _{A}'=-1$:

42 $$\begin{aligned} \hat{\rho }_{\mathfrak {E}|\kappa _{A}'=-1,\pmb {\mathbb {P}}}=\mathinner {|{\mathfrak {E}_{\kappa _{A}'=-1}^{\pmb {\mathbb {P}},\gamma _p}}\rangle }\mathinner {\langle {\mathfrak {E}_{\kappa _{A}'=-1}^{\pmb {\mathbb {P}},\gamma _p}}|} \otimes \bigg (\sum _{\kappa _B'\kappa _C'} ~ p(\kappa _B',\kappa _C'|\kappa _A'=-1,\pmb {\mathbb {P}},\gamma _p)\mathinner {|{\mathfrak {E}_{\kappa _B'\kappa _C'|\kappa _A'=-1}^{\pmb {\mathbb {P}},\gamma _p}}\rangle }\mathinner {\langle {\mathfrak {E}_{\kappa _B'\kappa _C'|\kappa _A'=-1}^{\pmb {\mathbb {P}},\gamma _p}}|} \bigg ); \end{aligned}$$

if $$\kappa _{A}'=1$$:

43 $$\begin{aligned} \hat{\rho }_{\mathfrak {E}|\kappa _{A}'=1,\pmb {\mathbb {P}}}=\mathinner {|{\mathfrak {E}_{\kappa _{A}'=1}^{\pmb {\mathbb {P}},\gamma _p}}\rangle }\mathinner {\langle {\mathfrak {E}_{\kappa _{A}'=1}^{\pmb {\mathbb {P}},\gamma _p}}|} \otimes \bigg (\sum _{\kappa _B'\kappa _C'} ~ p(\kappa _B',\kappa _C'|\kappa _A'=1,\pmb {\mathbb {P}},\gamma _p)\mathinner {|{\mathfrak {E}_{\kappa _B'\kappa _C'|\kappa _A'=1}^{\pmb {\mathbb {P}},\gamma _p}}\rangle }\mathinner {\langle {\mathfrak {E}_{\kappa _B'\kappa _C'|\kappa _A'=1}^{\pmb {\mathbb {P}},\gamma _p}}|} \bigg ). \end{aligned}$$

The same method explained above may be used to determine components of these density matrices in the $\{\mathinner {|{\Phi _{0}^{(B)}}\rangle },\mathinner {|{\Phi _{1}^{(B)}}\rangle }\}\otimes \{\mathinner {|{\Phi _{0}^{(C)}}\rangle },\mathinner {|{\Phi _{1}^{(C)}}\rangle }\}$ basis. The eigenvalues may then be used to calculate the second term in the Holevo bound.

### Postselection

We now demonstrate how the single point quantities may be used to calculate the postselected rate $R_{PS}$. The mutual information $I_{AB}$ may be found by integrating the single point mutual information $\tilde{I}_{AB}$

44 $$\begin{aligned} I_{AB}=\int p(\pmb {\mathbb {P}},\gamma _p) \ \tilde{I}_{AB}(\pmb {\mathbb {P}},\gamma _p) \ d\pmb {\mathbb {P}}~d\gamma _p \end{aligned}$$

Similarly we do the same for the Holevo information:

45 $$\begin{aligned} \chi =\int p(\pmb {\mathbb {P}},\gamma _p) \ \tilde{\chi }(\pmb {\mathbb {P}},\gamma _p) \ d\pmb {\mathbb {P}}~ d\gamma _p \end{aligned}$$

By defining the single point rate as $\tilde{R}=\tilde{I}_{AB}-\tilde{\chi }$. Thus the overall rate becomes:

46 $$\begin{aligned} R=\int p(\pmb {\mathbb {P}},\gamma _p) \ \tilde{R}(\pmb {\mathbb {P}},\gamma _p) \ d\pmb {\mathbb {P}}~ d\gamma _p . \end{aligned}$$

The postselection ensures the parties only use instances of the protocol where the single point rate is positive. Hence the postselected rate $R_{PS}$ becomes:

47 $$\begin{aligned} R_{PS}&=\int p(\pmb {\mathbb {P}},\gamma _p) \ \mathrm {max}\big [\tilde{R}(\pmb {\mathbb {P}},\gamma _p),0\big ] \ d\pmb {\mathbb {P}}~ d\gamma _p \end{aligned}$$

48 $$\begin{aligned}&=\int _{\Gamma } p(\pmb {\mathbb {P}},\gamma _p) \ \tilde{R}(\pmb {\mathbb {P}},\gamma _p) \ d\pmb {\mathbb {P}}~ d\gamma _p \end{aligned}$$

where $\Gamma$ denotes the region in which the single point rate is positive.

## Results

We now present the numerical results for the post-selected rate of the protocol. By utilising the relation $\tau =10^{-\gamma d}$ and setting $\gamma =0.02/ \mathrm {km}$ (equivalent to 0.2 db/km), which corresponds to state of the art fibre optics, the rate of the protocol is expressed in terms of distances (*d*) of the parties from the detector. In particular, we consider the symmetric configuration in which each of the parties is located the same distance from the detector. Other asymmetric configurations can be considered within the same framework, by mapping the distance of the user furthest away into the transmissivity of each incoming channel. Thus the results presented here represent the worst case scenario for any other asymmetric configuration of the parties.

**Figure 3:**
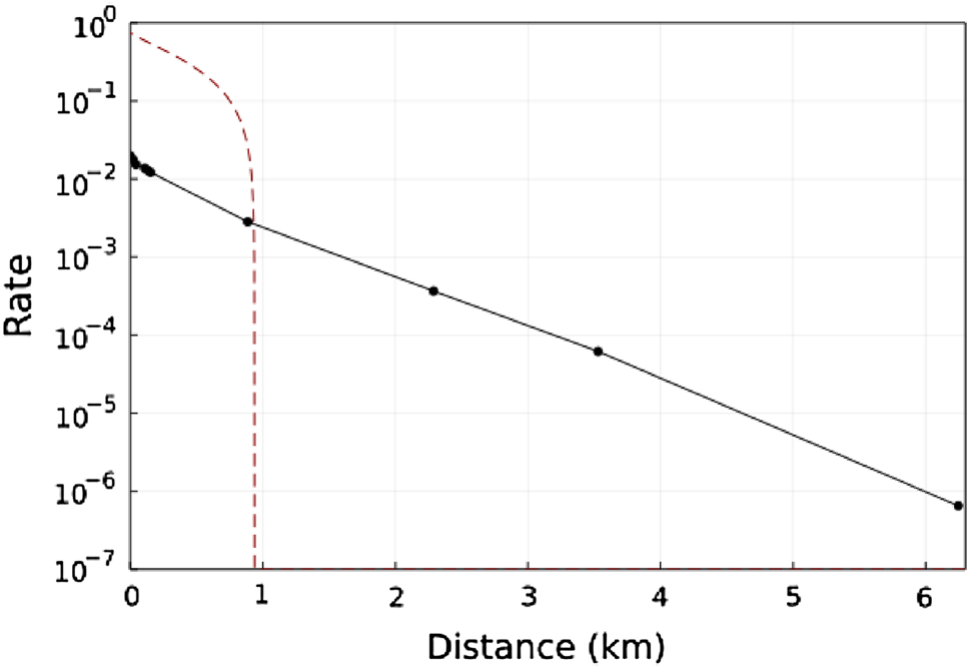
Post-selected rate of the protocol for the symmetric party configuration. Rate plotted with perfect detector efficiency and the variance in all prepared quadratures satisfy $\sigma _A=\sigma _B=\sigma _C=1$. The rate of the equivalent 3-party protocol from^39^ with optimised parameters, under a pure loss attack from is shown for comparison (red dashed line).

Figure 3 shows the rate-distance performance of the protocol in the asymptotic limit, assuming that a pure-loss attack is undertaken by Eve. We work with perfect detector efficiency and with the variance of each prepared quadrature $\sigma _{A}=\sigma _{B}=\sigma _{B}=1$ . We note that in general it may be possible to optimise the performance of the protocol over these parameters. Our results demonstrate that a positive rate can be maintained over a greater distance than in the corresponding 3-party case (shown for comparison in Fig. 3, albeit at the cost of lower rates at short distances). In particular the new protocol outperforms the equivalent protocol without postselection for distances greater than $\sim \, 1\,\mathrm {km}$.

## Conclusion

We have demonstrated a 3-party CV-MDI-QKD protocol that combines a generalised Bell detection with a postselection regime based on performing reconciliation on the signs of prepared quadratures of coherent states. We show that improved rate-distance performance is possible compared to the equivalent 3-party protocol without postselection, allowing a rate in excess of $10^{-4}$ bits per use at greater than $3\,\mathrm {km}$ and a positive rate for distances of up to $\sim \,6\,\mathrm {km}$. Our protocol also outperforms the equivalent protocol without postselection for distances greater than $\sim \,1\,\mathrm {km}$. Moreover since these protocols have exactly the same structure in terms of state preparation and the detector relay, it is possible to use one such relay to perform either protocol, choosing whichever will give the higher rate. That is, if the users are able to establish their distances from the detector, they choose whether or not to announce the absolute values of their quadratures and undertake postselection depending on whether or not this will produce a better rate. Whilst $\sigma _A,\sigma _B,\sigma _C$ are preset so any optimisation over these parameters must consider both protocols simultaneously it is still possible to retain the advantages of higher rate at shorter distances from the non-postselected protocol in addition to the improved long distance performance from our protocol.

The need to undertake a high-dimensional numerical integral given in Eq. (47), for a function that cannot be given in closed form (Eve’s conditional output state following the protocol $\hat{\rho }_{\mathfrak {E}|\pmb {\mathbb {P}},\gamma _p}$ has dimension $2^N$. Therefore for $N\ge 3$, including the tri-partite case considered in this paper, the eigenvalues of this state cannot be given in closed form. Therefore the entropy of the state and consequently the single point Holevo information $\tilde{\chi }$ can only be evaluated numerically for given values of the protocol’s parameters. This greatly complicates numerical integration in Eq. (47) as no explicit expression for the single point rate can be given. It is for this reason that our analysis is limited to pure loss attacks and three users, even though the analysis is readily extended to an arbitrary number of users and entangling cloner attacks.) to compute the post-selected key rate, limits our analysis to the 3-party case and pure-loss attacks. Nonetheless it may be possible to extend the study to the general *N* party case, maintaining the same structure of detector as in^39^ and considering entangling cloner attacks. Thus, our new protocol demonstrates that secure, multi-party conferencing can be achieved over improved distances, while retaining the security advantages of an MDI QKD protocol.

## Data Availability

The datasets used and analysed during the current study are available from the corresponding author on reasonable request.

## References

[1] Pirandola S, Wallden P (2020). Advances in quantum cryptography. Adv. Opt. Photon..

[2] Wootters WK, Zurek WH (1982). A single quantum cannot be cloned. Nature.

[3] Ekert AK (1991). Quantum cryptography based on Bell’s theorem. Phys. Rev. Lett..

[4] Quantum cryptography: Public-key distribution and coin tossing. In *Proceedings of IEEE International Conference on Computers, Systems and Signal Processing, Bangalore, India*, pp. 175–179 (1984).

[5] Bennett CH (1992). Quantum cryptography using any two nonorthogonal states. Phys. Rev. Lett..

[6] Stucki D, Walenta N, Vannel F, Thew RT, Gisin N, Zbinden H, Gray S, Towery CR, Ten S (2009). High rate, long-distance quantum key distribution over 250 km of ultra low loss fibres. New J. Phys..

[7] Pittaluga M, Minder M, Lucamarini M, Sanzaro M, Woodward RI, Li M-J, Yuan Z, Shields AJ (2021). 600-km repeater-like quantum communications with dual-band stabilization. Nat. Photon..

[8] Zhang Y, Chen Z, Pirandola S, Wang X, Zhou C, Chu B, Zhao Y, Xu B, Yu S, Guo H (2020). Long-distance continuous-variable quantum key distribution over 202.81 km of fiber. Phys. Rev. Lett..

[9] Joshi SK, Aktas D, Wengerowsky S, Loncaric M, Neumann SP, Liu B, Scheidl T, Lorenzo GC, Samec E, Kling L, Qiu A, Razavi M, Stipcevic M, Rarity JG, Ursin R (2020). A trusted node-free eight-user metropolitan quantum communication network. Sci. Adv..

[10] Dynes JF, Wonfor A, Tam WW, Sharpe AW, Takahashi R, Lucamarini M, Plews A, Yuan ZL, Dixon AR, Cho J, Tanizawa Y, Elbers JP, Greißer H, White IH, Penty RV, Shields AJ (2019). Cambridge quantum network. NPJ Quantum Inf..

[11] Solomons NR, Fletcher AI, Aktas D, Venkatachalam N, Wengerowsky S, Loncaric M, Neumann SP, Liu B, Samec icv, Stipčevič M, Ursin R, Pirandola S, Rarity JG, Joshi SK (2022). Scalable authentication and optimal flooding in a quantum network. PRX Quantum.

[12] Barrett J, Hardy L, Kent A (2005). No signaling and quantum key distribution. Phys. Rev. Lett..

[13] Schwonnek R, Goh KT, Primaatmaja IW, Tan EY, Wolf R, Scarani V, Lim CC (2021). Device-independent quantum key distribution with random key basis. Nat. Commun..

[14] Pironio S, Acin A, Brunner N, Gisin N, Massar S, Scarani V (2009). Device-independent quantum key distribution secure against collective attacks. New J. Phys..

[15] Braunstein SL, Pirandola S (2012). Side-channel-free quantum key distribution. Phys. Rev. Lett..

[16] Lo HK, Curty M, Qi B (2012). Measurement-device-independent quantum key distribution. Phys. Rev. Lett..

[17] Pirandola S, Ottaviani C, Spedalieri G, Weedbrook C, Braunstein SL, Lloyd S, Gehring T, Jacobsen CS, Andersen UL (2015). High-rate measurement-device-independent quantum cryptography. Nat. Photon..

[18] Tang G-Z, Li C-Y, Wang M (2021). Polarization discriminated time-bin phase-encoding measurement-device-independent quantum key distribution. Quantum Eng..

[19] Kwek L-C, Cao L, Luo W, Wang Y, Sun S, Wang X, Liu AQ (2021). Chip-based quantum key distribution. AAPPS Bull..

[20] Cui ZX, Zhong W, Zhou L, Sheng YB (2019). Measurement-device-independent quantum key distribution with hyper-encoding. Sci. China Phys. Mech. Astron..

[21] Pirandola S, Laurenza R, Ottaviani C, Banchi L (2017). Fundamental limits of repeaterless quantum communications. Nat. Commun..

[22] Laudenbach F, Pacher C, Fung C-HF, Poppe A, Peev M, Schrenk B, Hentschel M, Walther P, Hübel H (2018). Continuous-variable quantum key distribution with Gaussian modulation—the theory of practical implementations (Adv. Quantum Technol. 1/2018). Adv. Quantum Technol..

[23] Lucamarini M, Yuan ZL, Dynes JF, Shields AJ (2018). Overcoming the rate-distance limit of quantum key distribution without quantum repeaters. Nature.

[24] Chen JP, Zhang C, Liu Y, Jiang C, Zhang WJ, Han ZY, Ma SZ, Hu XL, Li YH, Liu H, Zhou F, Jiang HF, Chen TY, Li H, You LX, Wang Z, Wang XB, Zhang Q, Pan JW (2021). Twin-field quantum key distribution over a 511 km optical fibre linking two distant metropolitan areas. Nat. Photon..

[25] Wang S, Yin ZQ, He DY, Chen W, Wang RQ, Ye P, Zhou Y, Fan-Yuan GJ, Wang FX, Chen W, Zhu YG, Morozov PV, Divochiy AV, Zhou Z, Guo GC, Han ZF (2022). Twin-field quantum key distribution over 830-km fibre. Nat. Photon..

[26] Yin HL, Fu Y (2019). Measurement-device-independent twin-field quantum key distribution. Sci. Rep..

[27] Silberhorn C, Ralph TC, Lütkenhaus N, Leuchs G (2002). Continuous variable quantum cryptography: Beating the 3 dB loss limit. Phys. Rev. Lett..

[28] Symul T, Alton DJ, Assad SM, Lance AM, Weedbrook C, Ralph TC, Lam PK (2007). Experimental demonstration of post-selection-based continuous-variable quantum key distribution in the presence of Gaussian noise. Phys. Rev. A Atom. Mol. Opt. Phys..

[29] Wilkinson KN, Papanastasiou P, Ottaviani C, Gehring T, Pirandola S (2020). Long-distance continuous-variable measurement-device-independent quantum key distribution with postselection. Phys. Rev. Res..

[30] Lin J, Upadhyaya T, Lütkenhaus N (2019). Asymptotic security analysis of discrete-modulated continuous-variable quantum key distribution. Phys. Rev. X.

[31] Liu WB, Li CL, Xie YM, Weng CX, Gu J, Cao XY, Lu YS, Li BH, Yin HL, Chen ZB (2021). Homodyne detection quadrature phase shift keying continuous-variable quantum key distribution with high excess noise tolerance. PRX Quantum.

[32] Murta G, Grasselli F, Kampermann H, Bruß D (2020). Quantum conference key agreement: A review. Adv. Quantum Technol..

[33] Chen K, Lo H-K (2007). Multi-partite quantum cryptographic protocols with noisy GHZ States. Quantum Inf. Comput..

[34] Fu Y, Yin HL, Chen TY, Chen ZB (2015). Long-distance measurement-device-independent multiparty quantum communication. Phys. Rev. Lett..

[35] Zhao S, Zeng P, Cao W-F, Xu X-Y, Zhen Y-Z, Ma X, Li L, Liu N-L, Chen K (2020). Phase-matching quantum cryptographic conferencing. Phys. Rev. Appl..

[36] Cao X-Y, Lu Y-S, Li Z, Gu J, Yin H-L, Chen Z-B (2021). High key rate quantum conference key agreement with unconditional security. IEEE Access.

[37] Li Z, Cao XY, Li CL, Weng CX, Gu J, Yin HL, Chen ZB (2021). Finite-key analysis for quantum conference key agreement with asymmetric channels. Quantum Sci. Technol..

[38] Grasselli F, Kampermann H, Bruß D (2018). Finite-key effects in multipartite quantum key distribution protocols. N. J. Phys..

[39] Ottaviani C, Lupo C, Laurenza R, Pirandola S (2019). Modular network for high-rate quantum conferencing. Commun. Phys..

[40] Banchi L, Braunstein SL, Pirandola S (2015). Quantum fidelity for arbitrary Gaussian states. Phys. Rev. Lett..

